# Composite submental flaps in facial reconstructive surgery involving the zygoma and orbit

**DOI:** 10.1186/s40463-020-00468-9

**Published:** 2020-10-20

**Authors:** Usman Khan, Sebastian Haupt, Matthew Rigby, S. Mark Taylor, Martin Corsten, Jonathan Trites

**Affiliations:** 1 Faculty of Medicine, Dalhousie University, 1459 Oxford Street, Halifax, NS B3H 4R2 Canada; 2 Division of Otolaryngology – Head and Neck Surgery, Dalhousie University, Dickson Building, QEII Health Sciences Centre, 5820 University Avenue, Halifax, NS B3H 2Y9 Canada

**Keywords:** Submental island flap, Osteocutaneous submental island flap

## Abstract

**Background:**

The submental island flap (SIF) is a reliable option for reconstructing defects in the facial region and offers several advantages when compared to free-flap alternatives. While the reconstructive applications of the SIF have been demonstrated in the lower face, there are limited reports on its utility as a composite flap for reconstructing defects of the upper facial skeleton. To our knowledge, we report the first cases of composite (osteocutaneous) SIFs used for reconstruction of complex facial defects involving the zygoma and lateral orbit respectively.

**Case presentations:**

Three consecutive cases are presented. All were performed following resection of skin cancers with invasion of the upper facial skeleton. The first case was a 68-year-old male with a longstanding history of non-melanoma skin cancers who presented with a 7 cm recurrent basal cell carcinoma (BCC) with bicortical invasion of the left zygoma. The second case was an 88-year-old female with several squamous cell carcinomas (SCC), including a dominant 7.1 cm SCC on the right temple with orbital invasion. A third case was a 75-year-old immunosuppressed male with a 6.5 cm SCC of the right cheek with invasion of the orbit and zygoma following prior resection as well as high dose radiotherapy. The operative management of all cases involved harvesting the SIF on its vascular pedicle alongside the inferior portion of the mandible with rigid fixation to address the bony defects. The first case was robust throughout adjuvant radiotherapy with no flap complications after 2 year follow up. The second patient received adjuvant radiation therapy to an area that was previously radiated. Although the flap remained viable for a year, the patient experienced delayed soft tissue loss over the bony segment and eventual devitalization of the distal flap. The third case achieved a satisfactory result with no complications.

**Conclusions:**

Our case series outlines a unique application of the composite (osteocutaneous) submental island flap (SIF) for reconstruction of complex facial defects involving the upper facial skeleton. The osteocutaneous SIF should be used with caution in patients receiving adjuvant radiotherapy who have a history of previous radiation to the same or overlapping field.

**Graphical abstract:**

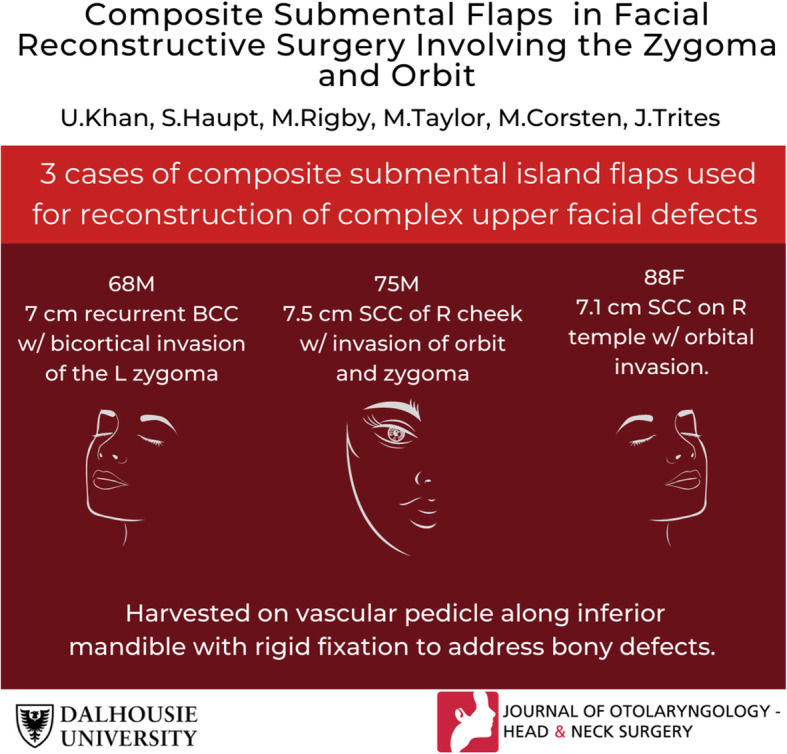

## Background

Facial reconstructive surgery is particularly challenging following resection of invasive cancers in the head and neck region. Complex facial defects involving bone often require free flaps from distant donor sites to optimize functional and aesthetic outcomes. Free flaps are chosen in these circumstances to provide robust areas of soft tissue with vascularized bone [1, 2]. However, free flaps require specialized equipment, microvascular expertise, increased health-care costs and long hospital stays [1, 2]. In many cases, regional flaps are sound alternatives to free flaps that utilize less health-care resources while maintaining excellent results [1, 2].

The pedicled submental island flap (SIF) is gaining popularity for its diverse applications in facial reconstructive surgery. The flap was first described by Martin et al. and is supplied by the submental vessels of the facial artery or retrograde flow from the angular artery [3, 4]. The SIF has demonstrated excellent results for reconstruction of defects involving the oral cavity, oropharynx, hypopharynx and lower face [1]. The flexibility of the vascular pedicle allows the SIF to extend to regions beyond the lower facial structures [4, 5]. However, the applications of SIFs in reconstruction of the upper face, especially with defects involving the facial skeleton remain extremely limited in the current literature.

A unique feature of the SIF is the ability to harvest the flap with the inferior portion of the mandible as a source of vascularized bone [4, 5]. This feature derives from the submental branch of the facial artery, which also gives off several unnamed penetrating vessels that supply the inferior mandibular component [4]. Previous injection studies have confirmed excellent vascularity through the bony component of the flap [4]. There are only few studies in the current literature that describe approaches for using the composite SIF to reconstruct defects involving bone outside the lower face. The majority of case reports using an osteocutaneous SIF in this region are limited to maxillary reconstruction [6–8]. A very recent study describes a series of eight patients who successfully underwent reconstruction of defects involving the maxilla, orbital rim or short zygomatic arch component with a composite SIF [8]. Another group has also previously reported on the osteomyocutaneous SIF for reconstruction of the inferior orbital rim [5]. Overall, the applications of the composite SIF beyond lower facial structures are limited in the literature, especially in cases of complex facial defects.

In the present series, we report three consecutive cases of composite (osteocutaneous) SIFs used for reconstruction of the upper facial skeleton. All cases required the resection of aggressive, recurrent skin cancers followed by reconstruction using the composite SIF. To our knowledge, we report the first cases of osteocutaneous SIFs used for reconstruction of complex facial defects involving the lateral orbit and zygoma respectively.

## Case presentations

### Case 1

**Fig. 1:**
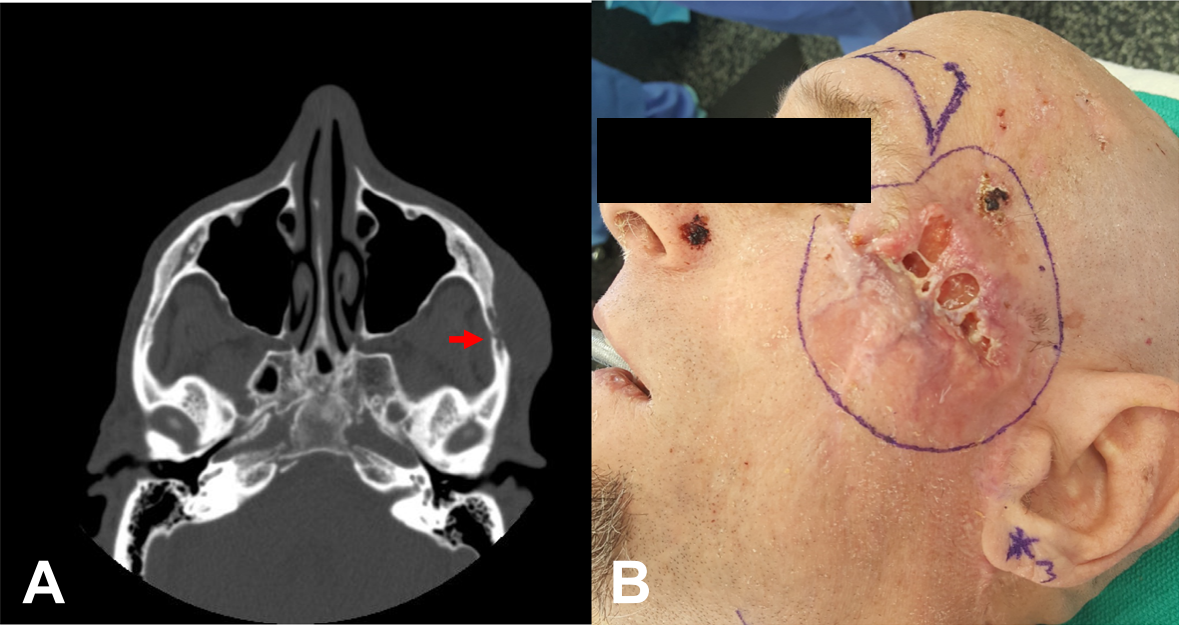
**a** Axial CT image revealing a 7 X 2.2 X 6 cm mass with extensive bony erosion of the left zygomatic arch (red arrow). **b** Preoperative digital image depicting a BCC that covers the full width of the left temple and a 6 mm BCC on the left nasal ala

A 68-year-old male was referred to our institution for surgical management of a large, 7 cm, recurrent basal cell carcinoma (BCC) involving the upper face. Clinical examination revealed an ulcerative lesion impinging on the left canthus and lateral orbit with extension to within a centimeter of the helical root posteriorly. Computed tomography (CT) confirmed extensive invasion of underlying tissues including bony invasion of the zygomatic arch and posterior body of the zygoma (Fig. 1).

A wide local excision of the primary tumor was performed including the entire temple from the left lateral canthus and orbit to the route of the helix and tragus. The tumor was found to penetrate extensively into the underlying tissue, and thereby required a limited parotidectomy with further dissection of the temporal fossa. Ultimately, the entire lateral canthus, large portion of the left lower eyelid, entire zygomatic bone, left lateral orbit and sections of mid-facial muscles were resected leaving a large facial defect.

**Fig. 2:**
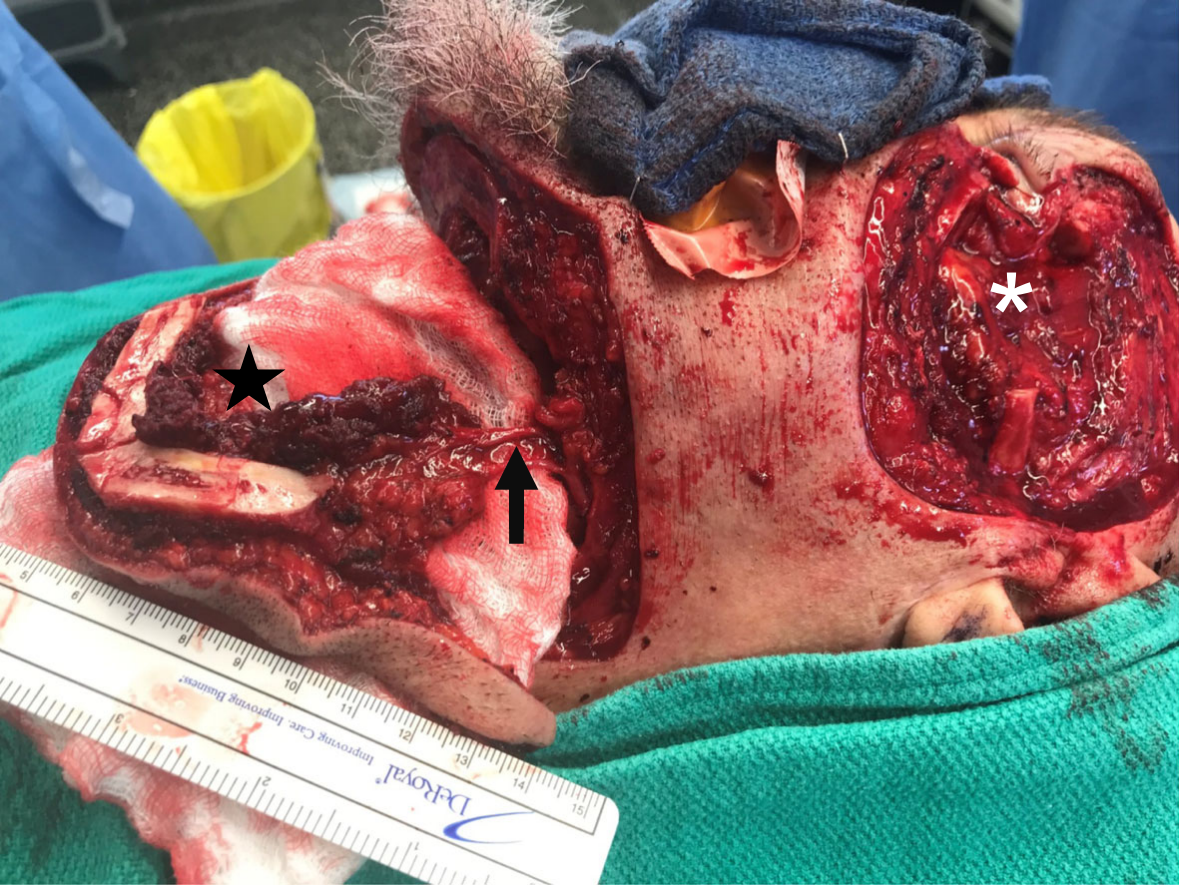
Intra-operative digital image outlining the defect after resection of the lateral orbit and zygomatic arch (*), vascular pedicle encompassing the submental artery and vein (arrow), and pedicled osteocutaneous flap involving the inferior mandible (star)

**Fig. 3:**
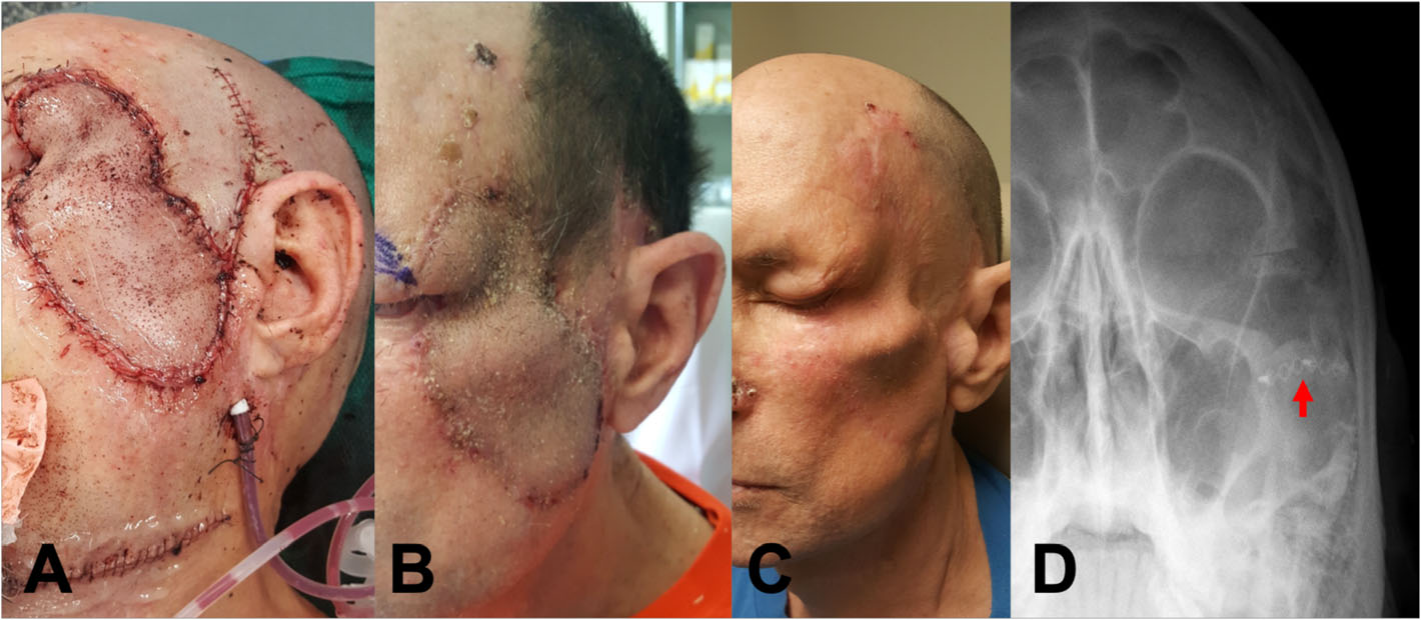
**a** Digital images of surgical site immediately after surgery, **b** 6-weeks post-operatively, **c** on 1-year follow up noting soft tissue loss around reconstructed zygoma after high dose adjuvant radiation, and **d** radiograph showing the site of rigid fixation (arrow)

A composite submental island flap (6 cm vertical X 8 cm transverse) was harvested with the inferior border of the mandible (1 cm vertical X 7 cm transverse) (Figs. 2 and 3). A level I neck dissection was performed with preservation of the submental branches of the facial artery and vein. The marginal mandibular branches of both facial nerves were identified and preserved during flap elevation. A perforator of the mylohyoid muscle supplying the mandibular segment was included alongside the left anterior digastric muscle to ensure adequate blood supply. The pedicle was completely dissected with a single submental artery and vein. Soft tissue and bone perfusion were confirmed with a doppler probe. The flap was transposed into the facial defect through a subcutaneous tunnel in the lower face. The zygomatic arch and lateral orbit were reconstituted with the vascularized mandibular bone using mini-plate fixation. The lateral canthus was reconstructed by suspending the lower lid remnant from the periosteum of the remaining orbit superiorly. The great majority of the skin defect was reconstructed with the SIF. However, a small superior temporal defect remained, and this was addressed with a local (superiorly based scalp) flap as well as a full thickness skin graft from the unused portion of the original submental flap.

The patient remains disease-free with no complications on 2-year follow up. The flap was robust throughout adjuvant radiotherapy. The patient required one revision procedure to address lower lid ectropion after radiation, as well as an unrelated BCC. The latter was done with a concurrent mid-brow lift to address a pre-existing brow ptosis and to provide a full thickness skin graft for the lower lid malposition.

### Case 2

An 88-year-old female was referred to our institution for surgical management of recurrent squamous cell carcinomas (SCC). Clinical and radiological examination identified a 7.1 cm right temple mass with extensive invasion of underlying tissues including the lateral orbit. The patient experienced diplopia and diminished mobility of the right eye. A head CT revealed a mass continuous with the lacrimal gland, lateral rectus muscle and globe with extension into the lateral orbital wall and roof.

**Fig. 4:**
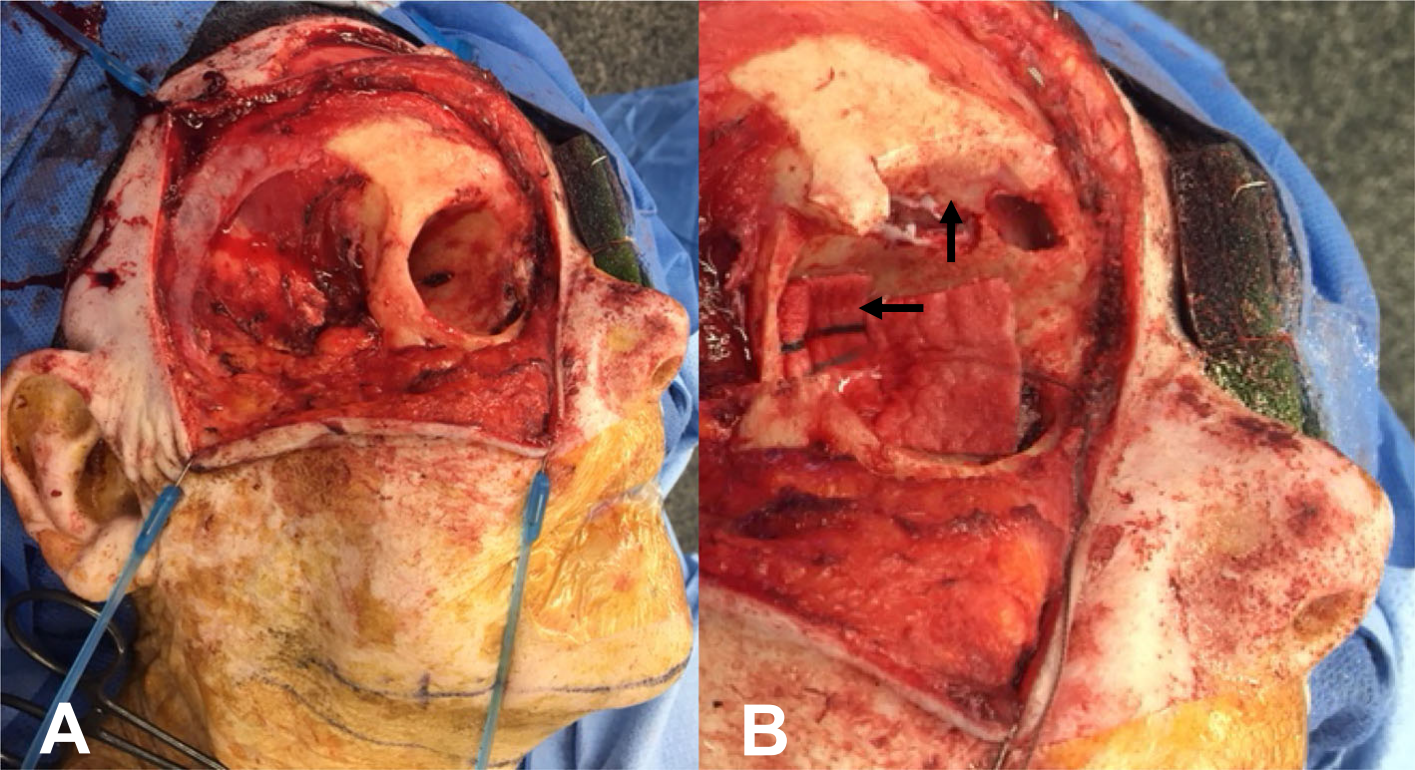
**a** Intra-operative digital images outlining the area of soft tissue resection and **b** regions of secondary orbital bone resection (arrows) with exposure of frontal dura and frontal sinus

**Fig. 5:**
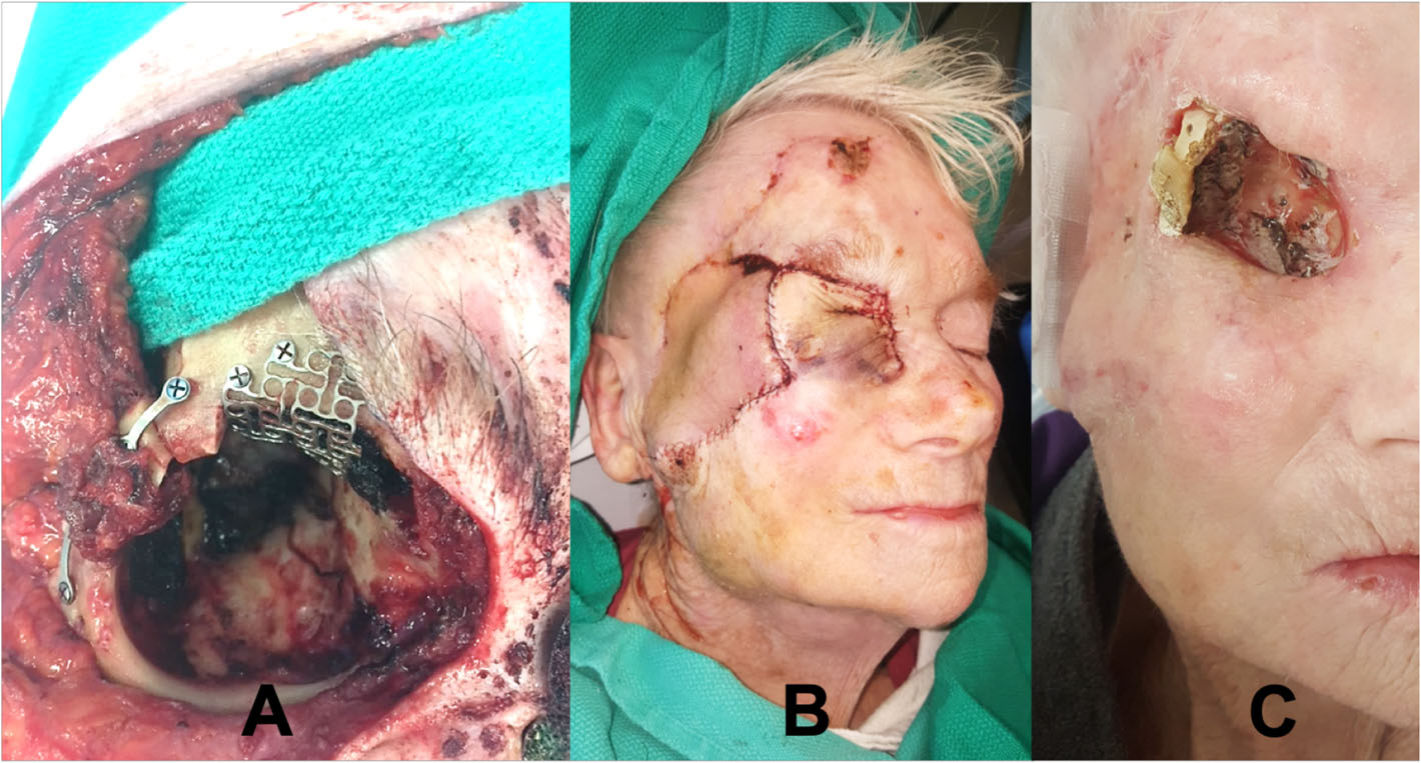
**a** Intra-operative digital image of mandible-to-orbit rigid fixation (skin paddle draped over green towel), **b** revision surgery (cheek flap) to address frontal sinus fistula before radiation treatment, and **c** image following radiation treatment outlining an area of soft tissue loss with exposure of bone and loss of distal flap

The tumor resection resulted in a large orbital and temporal defect. The orbital roof resection unfortunately led to a cerebral spinal fluid leak (Fig. 4). The dural defect was repaired successfully with layered temporalis fascia and titanium mesh that was cantilevered off the frontal bone and rigidly fixed. A submental island flap (20 cm transverse and 6 cm vertical) was utilized for reconstruction. The flap was harvested in conventional fashion with preservation of the facial vessels and marginal mandibular nerves. The lower border of the mandible was harvested with incorporation of the right mylohyoid muscle and anterior belly of the digastric muscle into the soft tissue component of the flap to protect both skin and bone perforators. The flap was delivered via a subcutaneous tunnel in the posterior cheek to reconstruct the facial defect and the mandibular component was used to reconstruct the right lateral orbit through rigid fixation (Fig. 5). The proximal skin of the SIF was deepithelialized to prevent burying of the epidermis subcutaneously and used as a full-thickness skin graft for a residual defect on the upper forehead.

The post-operative pathology confirmed clear resection margins. Although the flap was completely viable, radical adjuvant radiation therapy led to loss of overlying soft tissue over time (Fig. 5). It is important to note that the bony segment remained healthy and well-integrated with the zygomatic and orbital remnants well after radiation. Unfortunately, the loss of soft tissue caused exposure of the bone segment and eventual devitalization of the distal flap over a year after the initial surgery. The patient received a full thickness skin graft to cover the distal defect. She remains disease-free 29 months following surgery.

### Case 3

A 75-year-old male with a history of extensive cutaneous malignancies was referred to our institution for surgical management. His malignancies occurred in the context of chronic immunosuppression following kidney transplantation and insulin-dependent diabetes mellitus. Previous treatments had included 3 prior surgeries, 2 before radical radiation and one after as a salvage procedure involving radical parotidectomy and segmental resection of the right zygomatic arch and lateral orbit. Pathology showed an invasive 6.5 cm SCC with close margins and multiple positive regional nodes. A 3.5 cm recurrence was identified in the lower face at 6 month follow up.

**Fig. 6:**
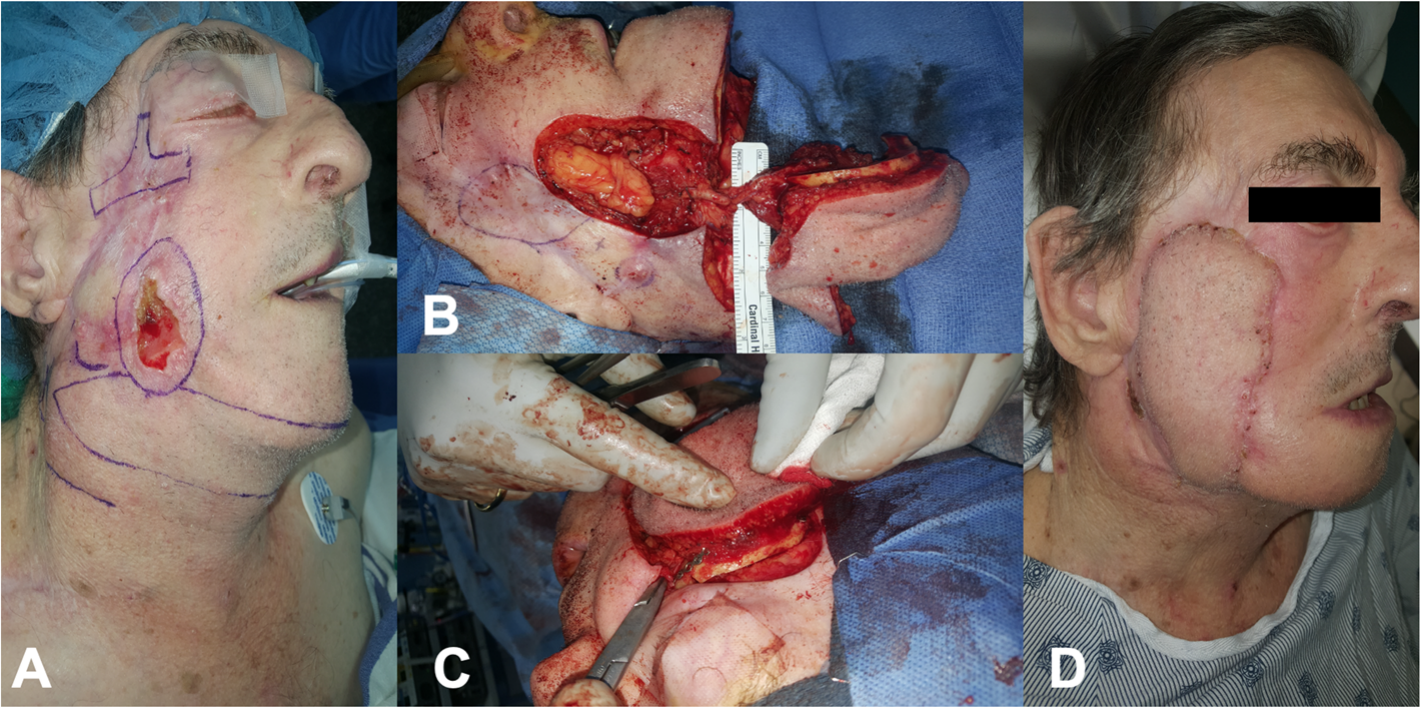
**a** Pre-operative digital image outlining the recurrent SCC in the lower face and the extensive zygomatic defect of the upper face, **b** Intra-operative digital image of the harvested osteocutaneous submental island flap including the vascular pedicle and inferior mandible. **c** Intra-operative digital image showing rigid fixation of the mandibular component of the flap, from the right lateral maxilla to the zygomatic root, **d** Post-operative digital image after one-month demonstrating a healthy flap, an excellent color match and improved cheek projection. Facial ptosis reflects previous radical parotidectomy

The patient underwent additional surgery with reconstruction of the SCC defect of the lower face as well as the zygomatic and temporal defect of the upper face (Fig. 6). The osteocutaneous submental flap was harvested as described in the previous cases with a large skin paddle measuring 22 cm and 6.5 cm in the transverse and craniocaudal planes respectively. Level IA and IB nodes were carefully identified and removed. The mobilized pedicle provided a substantial arc of rotation, affording a tension-free delivery of the flap to the surgical defect. With rigid fixation, the harvested mandibular segment bridged the gap from the lateral maxilla to the zygomatic root. A full thickness skin graft from the unused submental island skin paddle was utilized to reconstruct a 1.5 X 1.5 cm periauricular defect following resection of an unrelated SCC. The patient had an unremarkable post-operative course and was satisfied with the operative result.

## Discussion

The submental island flap (SIF) is an excellent option for reconstructing a variety of defects that extend beyond the lower facial region. Several studies have outlined practical advantages of the SIF, including safe dissection by residents in-training, enhanced outcomes when compared to radial forearm free flaps for similar defects, and excellent cosmetic results at both defect and donor sites [1, 3, 9–13].

When compared to free-flap alternatives, the SIF offers several advantages which include reduced operating time, lower health care costs and no requirement of microvascular expertise [1, 11]. The SIF can also be employed in elderly patients to avoid risks associated with free-flap reconstruction, as emphasized by our second and third cases on an 88-year-old female and 75-year-old male. From an operative standpoint, the SIF provides an aesthetically hidden donor site, excellent facial color match, wide axis of rotation and large area of well-vascularized soft tissue [1, 5]. Furthermore, the flap is thin and does not include bulky muscle tissue as is the case in other regional flaps [1]. These features of the SIF obviate the need for multiple pedicle divisions and sacrifice of functional muscle [1]. An osteocutaneous flap can also be harvested using the inferior mandible as a source of vascularized bone as demonstrated in our report [5, 11].

The limitations of the SIF include unwanted hair growth on the flap and the much more concerning potential for transfer of malignant upper neck nodes leading to recurrent cancer [1]. However, recurrence related to level I nodal seeding has not been our experience in over 80 regional transfers of this type. Patient selection is important: there is no increased risk of recurrence when the SIF is used in clinically node-negative patients [14]. We have used this flap in a number of patients with positive level I nodes, most of which were only identified with final pathology. A concerted effort is made to complete a thorough compartmental lymphadenectomy during flap harvest, and with this approach there have been no cases of disease recurrence. Our second case outlines a potential complication of combining the osteocutaneous SIF with adjuvant radiation therapy when the patient has previously received radiation to the same area. The loss of overlying soft tissue is detrimental to the bony component of the flap. Therefore, it is important to consider previous radiation exposure when assessing patients as candidates for the osteocutaneous SIF when adjuvant radiation treatment is anticipated.

To date, the reconstructive applications of the SIF have been focused predominantly on the lower face, oral cavity and pharynx. Our demonstration of the composite SIF to reconstruct facial defects involving the zygoma and lateral orbit has not been described previously.

## Conclusion

The composite (osteocutaneous) submental island flap has unique applications for reconstructing complex defects involving the upper facial skeleton. This flap can be used in patients requiring adjuvant radiation, and in those who have received prior radiotherapy and require salvage surgery. Caution should be used in patients who require more than one course of radiation to the same area. Further research is required to validate the effectiveness of the composite SIF in large patient cohorts.

## Data Availability

Not Applicable.
